# Nutritional Impact and Eating Pattern Changes in Schizophrenic Spectrum Disorders after Health Education Program on Symbiotic Dietary Modulation Offered by Specialised Psychiatric Nursing–Two-Arm Randomised Clinical Trial

**DOI:** 10.3390/nu14245388

**Published:** 2022-12-19

**Authors:** Alfonso Sevillano-Jiménez, Manuel Romero-Saldaña, María García-Rodríguez, Rafael Molina-Luque, Guillermo Molina-Recio

**Affiliations:** 1 Córdoba-South Community Mental Health Unit, Mental Health Clinical Management Unit, Reina Sofia University Hospital, C/Huelva s/n, 14013 Cordoba, Spain; 2 Department of Nursing, Pharmacology and Physiotherapy, University of Cordoba, Avd Menéndez Pidal s/n, 14004 Cordoba, Spain; 3 Lifestyles, Innovation and Health (GA-16), Maimonides Biomedical Research Institute of Cordoba (IMIBIC), Avd Menéndez Pidal s/n, 14004 Cordoba, Spain; 4 Department of Nursing and Nutrition, Biomedicine Sciences and Health Faculty, European University of Madrid, C/Tajo s/n, Villaviciosa de Odon, 28670 Madrid, Spain

**Keywords:** eating behaviour, diet, food, nutrition, nursing, schizophrenia spectrum, psychotic disorders, mental health

## Abstract

Background: The traditional therapeutic approach has perceived the role of nutrition as a minor intervention in psychiatry. The microbiota–gut–brain axis theory evidences the influence of dietary and nutritional patterns on mental health. Aims: To evidence the impact of dietary advice on increasing symbiotic intake on nutritional status and dietary habits in individuals with schizophrenia spectrum disorders. Methods: Randomised clinical trial (two-arm, double-blind, balanced-block, six-month intervention) in 50 individuals diagnosed with schizophrenia spectrum disorders. The control group received conventional dietary advice on an individual basis. A personal nutritional education programme was established in the intervention group (IG) to increase prebiotic and probiotic intake through dietary advice (dairy and fermented foods, green leafy vegetables, high-fibre fruit, whole grains, etc.). Data on nutritional status and dietary habits were collected (baseline and six months). The degree of dietary adherence to the recommended patterns was recorded weekly. Anthropometric parameters were also analysed monthly. Results: Finally, 44 subjects completed the follow-up. All participants exceeded the dietary reference intakes. The overall and intra-group analysis showed a statistically significant (*p* < 0.05) reduction in macro and micronutrient intakes with a closer approximation to the recommended dietary intakes, except for polyunsaturated fatty acids, oligosaccharides, polysaccharides and dietary fibre. After six months of intervention, statistical differences (*p* < 0.001) were found in all variables of the anthropometric profile in the IG, as well as an increase in the consumption of foods with a high symbiotic content (at baseline and six months). Likewise, a reduction in eggs, meat, fish, sugars and ultra-processed foods was evident, leading to significant intra-group differences (*p* < 0.05). Conclusions: Implementing conventional nutritional education strategies and specific nutritional advice with a symbiotic effect improves the dietary-nutritional profile in patients with schizophrenia spectrum disorders. Furthermore, it highlights the nutritional impact on mental health, stating itself as adjuvant therapy for physical health and lifestyle improvement.

## 1. Introduction

Historical evolution has evidenced the role of nutritional psychiatry as a minor intervention in the traditional therapeutic conception of Mental Health [1,2]. However, the advances in the last decade, mainly associated with the development of holobiont theory and metagenomics [2,3], as well as the detection of new dietary patterns of poor nutritional quality in different Western societies [1,3,4], have highlighted the influence exerted by the dietary and nutritional pattern on the functioning of the Central Nervous System (CNS) [5]. Furthermore, the possible mechanisms or aetiological pathways of psychiatric disorders have been established [2,3,5], especially in severe and long-term mental disorders (LTMD), such as schizophrenia [5]. Consequently, the “Microbiota–Gut–Brain Axis” concept has emerged. This term refers to the bidirectional communication pathway between the CNS, the gastrointestinal tract, and the microbiota (IM) [4,6]. Its determinant role in the organism’s normal functioning has been defined: development and maturation of the CNS, nutrition and metabolism, immune response or systemic inflammation [7,8,9,10,11]. Thus, according to low-grade systemic inflammation theory, when the state of dysbiosis appears, a cascade of pro-inflammatory agents can modify both the integrity and the permeability of enterocytes [3,9,10]. This response triggers the release of pro-inflammatory cytokines (tumour necrosis factor-alpha or interleukins type 6 or 1ß) [2,8,10], leading to synergies between inflammation, increased oxidative stress and energy imbalance [8]. These reactions result in homeostatic disturbances and neuropsychiatric dysfunction [9,11,12].

Dysregulation of the microbiota–gut–brain axis is determined by the acquisition of unhealthy lifestyles [1,5] based on poor nutritional quality dietary patterns and inadequate physical activity performance, which are especially common in LTMD [4,13,14,15]. Likewise, the current adoption of social distancing and home quarantine strategies established by the different governments within the global SARS-CoV-2 pandemic [16,17] promotes the acquisition of unhealthy habits in the vulnerable population, worsening previous pathogenic states [14].

### Background

Concerning mental problems, the evidence shows a high rate of disability and morbidity and mortality (up to 20% higher) [16,17,18,19], being especially significant in LTMD [3,5,15,18,19]. Furthermore, these figures are closely linked to the development of Metabolic Syndrome (MetS) [2,4,15,16,17,18,19], considered a determining factor in the patient’s physical health and tripling the incidence of cardio-metabolic diseases [17,18,19,20]. Finally, the main etiopathogenic determinants of MetS and the level of neuro-functional disability in the psychiatric population are linked to the characteristics of the disease, the exclusive psychopharmacological approach, and resistance to optimal care in terms of physical health and lifestyles [17,18,19,21].

Despite the magnitude and severity of the problem, interventions aimed at modifying nutritional patterns and eating behaviours play a minority role in the routine clinical practice of psychiatric healthcare professionals [4,15,16,21,22]. The evidence establishes high levels of malnutrition and the acquisition of unhealthy dietary habits in LTMD (far from the dietary reference standard) [4,15,22], based on the consumption of ultra-processed foods with high energy and glycaemic index and low consumption of fibre, fruit and vegetables [14,17,22,23,24]. Thus, the persistence of dietary patterns of low nutritional quality in the target population leads to a higher propensity for dysbiosis and, consequently, to a state of low-grade systemic inflammation. This state determines a higher risk of cardio-metabolic and psychopathological dysfunction that conditions the level of neuronal plasticity and cognitive performance [1,5,12]. 

In response to the evidence described above, there is currently a growing effort to develop and implement dietary interventions focused on modulating the gut microbiota in psychotic disorders through the use of “*psychobiotics*” [5,11,12,13] in the form of nutraceuticals. This term refers to a set of symbiotic substances (prebiotics and probiotics) whose administration leads to health benefits in psychiatric patients [5,10,11,25]. Probiotics include micro-organisms from the intestinal biota, which benefit the host when administered in adequate amounts (notably the genera Lactobacillus and Bifidobacterium, among others) [8,10,25,26]. On the other hand, prebiotics is non-digestible dietary fibre (fructo- and oligosaccharides, inulin or pectins) [2,5], which promote optimal growth and the development of probiotics in the gastrointestinal tract, reducing pathogenic microbiota [8,9,26].

In short, the future of the new models of care in Mental Health is determined by the approach to nutritional factors and the detection of unhealthy dietary habits [1,6,10,23,24]. This fact is conditioned by the appropriate use of nutritional counselling in the multifactorial approach to the psychiatric patient, representing the cornerstone in achieving optimal and cost-effective health outcomes for the health system [27,28].

For all the above, this study aimed to evaluate the impact of nutritional counselling to follow a high-symbiotic diet in patients diagnosed with a schizophrenia spectrum disorder and in the context of confinement and social restriction due to the SARS-CoV-2 pandemic.

## 2. Materials and Methods 

### 2.1. Study Design

**Figure 1:**
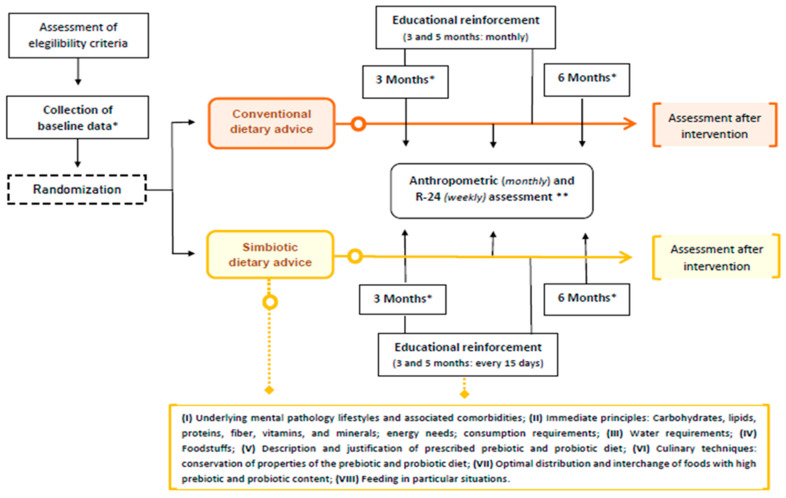
Study Design. * Data collected at baseline and six months of intervention: (1) Nutritional profile. ** Data collected at baseline and monthly during the intervention: (1) Anthropometric data (weight, height, Body Mass Index—BMI, waist circumference and waist-to-height ratio—WHtR-); (2) R-24 (weekly determination of foods with high symbiotic content in adherence to established dietary plan).

A 6-month, double-blind, two-arm, parallel-arm, balanced-block, randomised clinical trial was conducted on patients diagnosed with schizophrenia spectrum disorders (without distinction by type). The study design is shown in Figure 1.

### 2.2. Population Eligibility Criteria

The sample was selected from the referral Psychiatry Service from June 2020 to February 2021. Inclusion criteria were: (1) patients diagnosed on the spectrum of schizophrenia (without distinction by type), according to criteria DSM-5 and/or ICD-11; (2) age between 18–65 years; (3) absence of gastrointestinal comorbidity that contraindicates the use of prebiotics and/or probiotics (intolerance, explosive diarrhoea, acute abdominal pain, etc.); (4) to show clinical stability for six months before the beginning of the study (absence of psychiatric hospitalisation, maintenance of the level of functionality, and lack of social and occupational absenteeism); (5) to manifest agreement to participate in the study and to sign of informed consent. Reasons for exclusion of participants were: (1) suffering from a somatic or neurocognitive condition that prevented participation and collaboration in compliance with the protocol; (2) standardised dietary planning not modulated by the study population (catering, institutional or collective feeding, etc.); (3) refusal to participate in the study.

### 2.3. Sample Size

The researchers estimated a 22 individuals sample size to assess the efficacy of the intervention (11 for the control group (CG) and 11 for the intervention group (IG), with a power of 80% and safety of 95%, expecting a risk/prevalence difference of 63% post-intervention [29]. Finally, the sample was increased by more than 50% to minimise the effect of possible losses, obtaining a final size of 50 individuals (25 for the CG and 25 for the IG). Randomisation was performed according to the anthropometric analysis results, allowing the prevalence of MetS in both groups to be balanced.

### 2.4. Intervention

The CG consisted of those participants who received conventional dietary advice [30] on an individual basis. Similarly, the IG intervention was developed individually through intensive nutritional intervention [31] (designed and supervised by registered dietitians) from specialised nurses on psychiatric care and based on counselling for the increasing consumption of food with high prebiotic and probiotic content. In both allocation groups, visually supportive educational resources were used during the consultations [32]. The study was initiated with focus groups that improved the set dietary-nutritional intervention, ensuring its correct adaptation according to the study population. The research project was also presented to the referred psychiatry service staff. Subsequently, a 6-month dietary-nutritional education programme was implemented, associated with two months of educational reinforcement every 15 days for the IG and monthly for the CG. During the intervention phase, the anthropometric status (weight, body mass index—BMI, waist-to-height ratio—WHtR-, and waist circumference) was determined monthly, as well as the dietary-nutritional pattern using a validated Food Frequency Questionnaire (FFQ) for the Spanish population [33], focusing on those food groups and main dishes with the most significant symbiotic impact, at baseline and six months after the intervention. Finally, to assess the degree of adherence to the established dietary plan, a weekly record was kept of the main dishes and foods consumed with a prebiotic and probiotic effect (fermented foods, whole grains, green leafy vegetables, fruit, etc.).

### 2.5. Data Analysis 

The data were described using means and standard deviation for quantitative variables and frequencies and percentages for qualitative variables. The Kolmogorov-Smirnov test was used to assess normality in quantitative variables. The Student’s t-test for paired data, Mann-Whitney U test and repeated-means ANOVA were used to analyse the relationship between quantitative variables. Similarly, the association between qualitative variables was determined by Chi-square (Fisher or Yates corrections) and McNemar’s test. Non-parametric versions of the tests described above were performed if homoscedasticity criteria were not met. A probability of alpha error of less than 5% (*p* < 0.05) and a confidence interval of 95% were accepted during the analysis. Finally, SPSS (version 25.0) and EPIDAT (version 4.2) software were used for computing these tests. Similarly, the Nutriplato 2.0 (version 4.6) software tool [34] was used in the FFQ analysis.

## 3. Results

**Figure 2:**
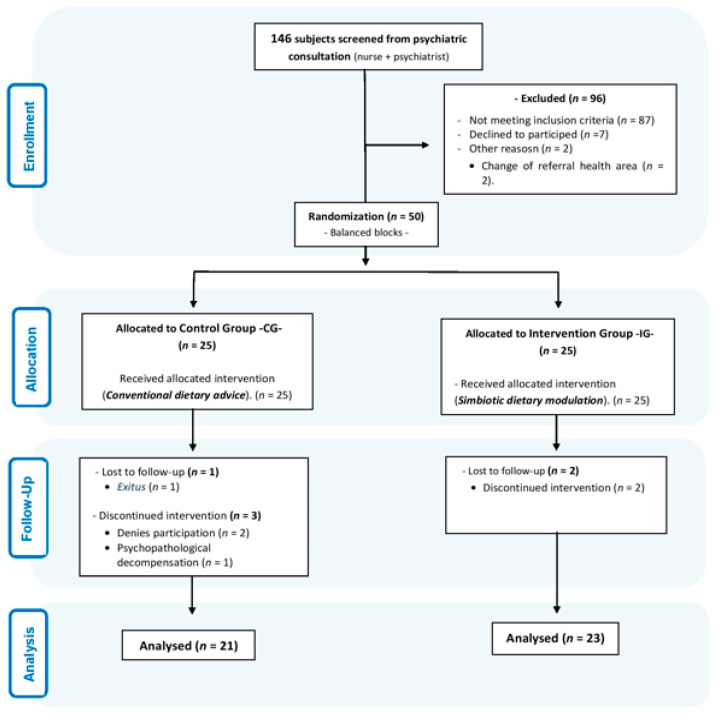
CONSORT flow diagram.

During recruitment, the eligible population was 50 subjects. Six participants were excluded during the intervention phase, resulting in 21 subjects in the CG and 23 in the IG. The flow chart of the participants is shown in Figure 2. 

Thirty-two men (72.7%) and 12 women (27.3%) participated, with 49.2 ± 11.9 years on average. The primary psychiatric diagnosis was schizophrenia [37 (84.1%)], with a mean duration of illness of 21.6 ± 12.4 years. Drug use was reported by 29 smokers (65.9%), ten subjects who used cannabis (22.7%) and 6 participants who reported drinking alcohol (13.6%) regularly. Regarding the number of subjects with associated cardio-metabolic risk factors, 17 subjects showed dyslipidaemia (38.6%), ten high blood pressure (22.7%), and seven suffered from diabetes mellitus (15.9%). Moreover, 27 participants (61.4%) knew how to cook and were responsible for it. 

Finally, the baseline analysis of the dependent variables showed significant differences in sugars and ultra-processed products and the mean calculation (weekly, monthly and quarterly) of the main foods and dishes consumed with high symbiotic value. Table 1 and Table 2 show the baseline characteristics of the independent and dependent variables, respectively, showing homogeneity between the two allocation groups.

Table 3 shows the changes in outcome variables at baseline and six months of intervention in CG and IG, respectively. The overall analysis showed that almost all participants significantly exceeded the Recommended Daily Allowances (% RDA), except for the vitamin D variable, were lower than recommended throughout the intervention phase. Likewise, the overall analysis showed a significant improvement (*p* < 0.05) in all macronutrient and micronutrient profile variables, except for polyunsaturated fatty acids, oligosaccharides, polysaccharides, dietary fibre, copper, manganese, biotin, ascorbic acid and vitamin D.

Similarly, the overall analysis of weekly, monthly and quarterly records of consumption of foods with high symbiotic content showed statistically significant differences, with a subsequent decrease and increase in consumption coinciding with the implementation of social distancing and confinement measures in the SARS-CoV-2 era. 

Regarding the intra-group analysis of macronutrients between the CG and IG, we observed concordance with those results obtained from the global analysis, obtaining statistically significant differences in all variables, except for polyunsaturated fatty acids oligosaccharides, polysaccharides and dietary fibre. However, the intra-group analysis of micronutrients showed statistically significant differences in the IG for phosphorus, sodium, iron, zinc, thiamin, vitamin B12 and vitamin E. 

Similarly, in terms of weekly consumption by food group in the IG, there was a reduction in protein consumption of eggs, meat and fish, and sugars and ultra-processed foods, compared to the increase obtained in the CG. This fact is related to the non-statistical significance at the global level. In addition, there was an increase in the consumption of dairy products, legumes and cereals between the two allocation groups and a decrease in the intake of fruit, vegetables and greens. However, these variations were not significant. Finally, anthropometric variables improved significantly (*p* < 0.001) in the IG, while waist circumference increased in the CG. These modifications did not lead to significant differences in the number of antipsychotics and dosage prescribed. 

Regarding the global inter-group analysis at baseline and at six months of intervention, except for the improvement observed in the consumption of sugars and ultra-processed food (*p* < 0.05) in IG, no statistically significant results were shown for the rest of the dependent variables.

## 4. Discussion

This study focuses on the nutritional impact of a high symbiotic dietary modulation throughout health education intervention by specialised psychiatric nurses in patients suffering from schizophrenia spectrum disorder, reducing macro and micronutrient intake towards a closer approximation to the % RDA between allocation groups. However, the findings confirm the results described by Gill R et al. (2021). They found that implementing an intensified educational approach does not yield significant benefits compared to a conventional dietary-nutritional intervention in schizophrenic disorders [24]. 

On the other hand, from our perspective, we consider that the statistically significant intra-group differences are a reason for not reaching inter-group statistical results, a condition supported by the significance reached for the overall analysis of the group. However, the results obtained reflect a clinically significant trend toward healthier nutritional patterns in the IG, compared to the increased consumption of ultra-processed, higher energy and higher glycaemic index foods in the CG [4,15,16,35,36], a highly prevalent condition in the target population (*p* < 0.05) [20,32]. The scientific evidence supports and clarifies the results obtained, with an increase in dietary habits of low nutritional quality in psychotic disorders (up to 60%) [21,32,36]. These patterns exceed % RDA [35,36] and are characterised by a higher intake of refined carbohydrates, saturated fats, sodium and phosphorus, as well as a lower intake of vitamin D, calcium, potassium, iron, polyunsaturated fatty acids, fruit and vegetables and, therefore, lower intake of dietary fibre (among others) [1,4,15,16,22,35,36,37,38,39,40,41,42]. In this regard, according to Gill R et al. (2021), Stefańska E et al. (2019) and Kowalski K et al. (2022), low adherence to Mediterranean dietary patterns leads to a higher propensity for nutritional deficiencies [24,36,41] and, consequently, a higher risk of exacerbation of underlying cardio-metabolic and neuropsychiatric disorders [39,40,41]. 

Undoubtedly, the improvement of the anthropometric profile (in all variables) and, therefore, the significant decrease in the risk of MetS in the GI (*p* < 0.05) after intensive dietary advice with high prebiotic and probiotic content is noteworthy. Similar findings were obtained by Sugawara et al. (2018) and Caemmerer et al. (2012) [29,42]. Again, the results presented in the present study support the meta-analysis developed by Teasdale et al. (2017), showing that non-pharmacological interventions focused on improving the dietary-nutritional pattern are established as coadjuvant therapies for metabolic abnormalities [43], relevant to improving the lifestyles of the target population [1,4,15,23,35]. Concerning the level of compliance and results obtained in both allocation groups, it is essential to highlight the contextual framework of the global SARS-CoV-2 pandemic in which this clinical trial was conducted. Solé et al. (2021) indicated that most preliminary studies during the current pandemic have focused on psychological distress in the general population [14], with limited evidence regarding the dietary pattern followed in patients with schizophrenia spectrum disorders. Likewise, the particular vulnerability of the target population in this context of confinement and the global pandemic [14,16,17] stands out. This population has limited the acquisition of coping strategies, which has encouraged the development of unhealthy lifestyles [14,15,16,17,23,39], where hypercaloric dietary patterns and the restriction of physical activity stand out [23,24,40,41]. For these reasons, the effectiveness of the intervention may have been reduced. Thus, according to Stefánska et al. (2019), Stefánska et al. (2017), and Cheikl et al. (2021), poor sun exposure during states of confinement and the characteristics of schizophrenic disorder in terms of social restriction has made it challenging to achieve optimal vitamin D results in line with % RDA [35,36,40].

As established by Costa et al. (2019) and Giannouli (2017), the high prevalence of morbidity and mortality in LTMD is not only determined by the nutritional outcome and dysmetabolic status but also by the aetiological condition that derives from it [23,44]. In this sense, cultural, cognitive-emotional or spiritual factors stand out as aspects to be considered when elucidating which ones behave as protective or risk agents in the prediction of morbidity and mortality in the psychiatric population [44,45], especially in contexts of social restriction [14,17,40].

Thus, risk behaviours associated with the consumption of intoxicants, such as alcohol, tobacco or cannabis (among others), are a proven condition related to the factors above (cultural, cognitive, etc.) [44], with an impact on unhealthy lifestyles [23,28,39,40] and the development of disruptive behaviours [44,45]. However, despite the particular vulnerability of the psychiatric population to states of confinement [14,17,18], the latter has been postulated as a protective factor against the development of harmful habits with marked social content, such as substance consumption, among which alcohol consumption stands out. This fact has made it possible to minimise the potential impact of alcohol intake on the results obtained regarding the nutritional and cardio-metabolic profile.

However, despite the significant difficulties of intervention in the schizophrenic population [1,17,32], this study highlights the feasibility of high-symbiotic dietary intervention on cardio-metabolic health and marked improvement of the nutritional profile, different from the current evidence available in confinement settings [16,18,24,38]. Longitudinal studies are needed to demonstrate the impact of hygienic-dietary measures on the macro and micronutrient profile in the psychiatric population [17,41].

The available evidence shows that clinical trials with dietary approaches in the absence of psychopharmacological treatment are limited [38], show marked heterogeneity and lack methodological rigour [5,10]. However, as Stefánska et al. (2019) and Dabke et al. (2019) point out, the association of the individual nutritional programme with a symbiotic approach may have a high synergistic impact on the improvement of dysmetabolic states [35,46], a highly prevalent condition in the population studied [17,18,20]. 

Finally, according to Balanzá (2017), the role of advanced practice nurses stands out as the cornerstone of the multidisciplinary approach and the main person responsible for the dietary advice offered [5]. Likewise, psychiatric nurses are the leading active players in the emotional and cognitive regulation associated with the dietary patterns established as part of the care provided to the psychiatric population [28,32].

### Limitations

The main limitations of this study are related to the sample size and the participants’ loss or lack of cooperation during the intervention phase. However, this limited and heterogeneous sample size could explain the scarce significant differences between macro and micronutrient profile variables and weekly consumption by food group. Thus, significant intra-group differences meant that no inter-group statistical results were achieved. Finally, since the authors followed the usual procedures for sample size estimation, it is possible that the risk/prevalence difference between IG and CG was overestimated (63%).

The results obtained may be linked to the difficulty of using FFQ in assessing the dietary-nutritional pattern. This fact is based on the low degree of dietary knowledge and food responsibility, psychopathological state and, therefore, possible associated neurocognitive impairment in the population under study, which may result in overestimated data relating to the RDAs [1,35].

Likewise, the non-inclusion of biochemical parameters in this manuscript may be a potential limitation, preventing the comparison of nutritional and cardio-metabolic results (blood glucose, lipid profile, etc.). The reason for not including these data was that the main objective was to assess the intervention’s efficacy in modifying this population’s dietary patterns with such particular characteristics. However, readers interested in this type of information can consult a recent paper in which these results have been included [47].

It would be relevant to know the psychopathological impact of increased consumption of prebiotics and probiotics on psychiatric disorders, of particular interest in LTMD. To this end, using Visual Evoked Potentials (VEP) is an effective diagnostic technique in analysing the neurophysiology of subjective disorders and interruption of functional recovery in psychopathological worsening, common in schizophrenia [48]. Unfortunately, this test could not be applied during the development of the trial, so subsequent studies that included it could provide more information on this vital topic.

It is essential to note that this study was conducted during the SARS-CoV-2 pandemic, making it difficult to achieve the proposed intervention, especially in acquiring and strengthening healthy lifestyles. Furthermore, it is necessary to consider the inherent characteristics of the subjects under study, a population that is particularly vulnerable to change, especially in a context of confinement and a global pandemic [14,17]. 

Finally, the scarcity of existing research on nutritional patterns and dietary habits in subjects diagnosed with schizophrenia makes it difficult to contrast the results obtained in different healthcare settings.

## 5. Conclusions

The development of a dietary-nutritional education programme in patients diagnosed with schizophrenia spectrum disorders and based on dietary advice by psychiatric nurses has not shown significant differences from conventional health education models, both being proposed as effective interventions in improving the nutritional pattern and dietary habits of the population under study. However, implementing a dietary-nutritional intervention with a high symbiotic content improves cardio-metabolic outcomes effectively in a global pandemic such as SARS-CoV-2. Furthermore, despite the inherent lifestyle dysfunctionalities of the target population, prebiotics and probiotics have been shown to offer a relevant and promising solution in different settings. Finally, further studies with larger sample sizes and outside the context of pandemics and confinement are needed to assess better the efficacy of these interventions.

## Figures and Tables

**Table 1:** Sample characteristics (independent variables): Baseline.

Variables		Total (*n* = 44)	Control Group (*n* = 21)	Intervention Group (*n* = 23)	*p*
***Socio-demographic variables***					
Sex					
	Men	32 (72.7%)	14 (31.8%)	18 (40.9%)	0.388
	Women	12 (27.3%)	7 (15.9%)	5 (11.4%)	
Age (years)		49.2 (11.2)	48.8 (13.8)	49.5 (10.1)	0.897
Household composition					
	Individual	12 (27.3%)	5 (11.4%)	7 (15.9%)	0.893
	Horizontal	3 (6.8%)	1 (2.3%)	2 (4.5%)	
	Complete	3 (6.8%)	1 (2.3%)	2 (4.5%)	
	Family home	7 (15.9%)	4 (9.1%)	3 (6.8%)	
	Supervised flat	19 (43.2%)	10 (22.7%)	9 (20.5%)	
Economic level					
	High	6 (13.6%)	3 (6.8%)	3 (6.8%)	0.651
	Medium	26 (59.1%)	11 (25%)	15 (34.1%)	
	Low	12 (27.3%)	7 (15.9%)	5 (11.4%)	
Education level					
	Uneducated	4 (9.1%)	2 (4.5%)	2 (4.5%)	0.590
	Primary	19 (43.2%)	11 (25%)	8 (18.2%)	
	Secondary	17 (38.6%)	7 (15.9%)	10 (22.7%)	
	University	4 (9.1%)	1 (2.3%)	3 (6.8%)	
Area of residence					
	Urban	38 (86.4%)	18 (40.9%)	20 (45.5%)	1.00
	Rural	6 (13.6%)	3 (6.8%)	3 (6.8%)	
***Clinical Variables***					
Psychiatric diagnosis					
	Schizophrenia	37 (84.1%)	19 (43.2%)	18 (40.9%)	0.419
	Schizoaffective Disorder	5 (11.4%)	1 (2.3%)	4 (9.1%)	
	Delusional Disorder	2 (4.5%)	1 (2.3%)	1 (2.3%)	
Duration of illness (years)		21.6 (12.4)	22.5 (12.6)	20.9 (12.5)	0.715
Age at first hospitalisation (years)		31.4 (11)	31.4 (11.4)	31.4 (10.7)	0.572
Consumption of toxics					
	No	15 (34.1%)	5 (11.4%)	10 (22.7%)	0.169
	Yes	29 (65.9%)	16 (36.4%)	13 (29.5%)	
Type of toxics					
	Alcohol	6 (13.6%)	3 (6.8%)	3 (6.8%)	0.775
	Tobacco	29 (65.9%)	15 (34%)	14 (31.8%)	
	Cocaine	3 (6.8%)	1 (2.3%)	2 (4.5%)	
	Opioids	2 (4.6%)	1 (2.3%)	1 (2.3%)	
	Amphetamines	3 (6.8%)	2 (4.5%)	1 (2.3%)	
	Cannabis	10 (22.7%)	5 (11.6%)	5 (11.3%)	
Cardio-metabolic condition					
	No	24 (54.5%)	11 (25%)	13 (29.5%)	0.783
	Yes	20 (45.5%)	10 (22.7%)	10 (22.7%)	
Type Cardio-metabolic condition					
	AHT	10 (22.7%)	6 (13.6%)	4 (9.1%)	0.407
	DM	7 (15.9%)	5 (11.3%)	2 (4.5%)	
	Hyperlipemia	17 (38.6%)	8 (18.1%)	9 (20.4%)	
***Therapeutic Variables***					
Reason for Change: Antipsychotic Treatment					
	Unchanged	31 (70.5%)	16 (51.6%)	15 (48.4%)	0.660
	Lack of efficiency	5 (11.4%)	1 (2.3%)	4 (9.1%)	
	Tolerability/safety issues	2 (4.5%)	1 (2.3%)	1 (2.3%)	
	Patient’s own choice	3 (6.8%)	1 (2.3%)	2 (4.5%)	
	Other: Clinical improvement	3 (6.8%)	2 (4.5%)	1 (2.3%)	
***Tolerability and Modulation of Dietary and Nutritional Patterns***					
Culinary knowledge and food responsibility					
	Can cook and he/she is in charge of it	27 (61.4%)	9 (20.5%)	18 (40.9%)	0.004
	Can cook but he/she is not in charge of it	6 (13.6%)	2 (4.5%)	4 (9.1%)	
	Cannot cook and he/she is not in charge of it	11 (25%)	10 (22.7%)	1 (2.3%)	

AHT: Arterial hypertension; DM: diabetes mellitus.

**Table 2:** Sample characteristics (dependent variables): Baseline.

Variables	Total (*n* = 44)	Control Group (*n* = 21)	Intervention Group (*n* = 23)	*p*
***Macronutrients*** (RDA)				
Energy (%)	177.4 (48.4)	182 (47.3)	173.2 (50.1)	0.329
Proteins (g)	432 (152.5)	443.2 (159.6)	421.9 (148.6)	0.597
Lipids (g)	207.8 (63.1)	212.4 (68.2)	203.5 (59.4)	0.716
Saturated fatty acids (g)	392.3 (222.2)	414.9 (260.6)	371.7 (183.9)	0.698
Monounsaturated fatty acids (g)	147.7 (60.9)	140.9 (63.6)	154 (59)	0.613
Polyunsaturated fatty acids (g)	138.9 (103.7)	145.1 (131.3)	133.2 (72.8)	0.716
Cholesterol (mg)	247.2 (135.8)	224.6 (70.3)	267.9 (175)	0.787
Carbohydrates (g)	159.6 (53.4)	161.8 (48.2)	157.5 (58.7)	0.518
Oligosaccharides (g) Polysaccharides (g)	303.4 (205.7)	342.7 (203.8)	267.5 (205.2)	0.088
	123.2 (74.6)	139.8 (87.2)	108 (58.8)	0.378
Fibre (g)	215.1 (124.8)	196 (130.5)	232.5 (119.5)	0.245
***Micronutrients*** (RDA)				
Ca (mg)	181.1 (68.8)	194.1 (64.7)	169.2 (71.2)	0.124
Mg (mg)	292.8 (95.4)	285.3 (88.8)	299.7 (102.6)	0.823
P (mg)	352.9 (107.7)	372.7 (123.5)	334.8 (89.9)	0.209
Na (mg)	310.8 (93.6)	320.9 (112.4)	301.5 (73.7)	0.630
K (mg)	236 (83.8)	232.4 (90.2)	239.2 (79.5)	0.664
Fe (mg)	216 (70.5)	211.1 (64.1)	220.5 (77.1)	0.953
Cu (mg)	173.7 (91.2)	170 (118.9)	177 (58.1)	0.184
Zn (mg)	260.4 (101.3)	251.2 (83.4)	268.7 (116.6)	0.916
Mn (mg)	714.7 (595.6)	575.9 (360.2)	841.3 (735)	0.264
I (ug)	252.7 (102.1)	278.4 (120.9)	229.2 (76.7)	0.177
Se (mg)	501.4 (229)	471 (203)	529.2 (251.7)	0.518
Thiamine (mg)	261.4 (81.4)	261 (74.1)	261.7 (89.2)	0.769
Riboflavin (mg)	289.2 (92.7)	299.5 (92.1)	279.8 (94.4)	0.226
Niacin (mg)	388.5 (116.3)	392.7 (113)	384.7 (121.6)	0.503
Pantothenic acid (mg)	97.3 (44.6)	103.6 (46.9)	91.6 (42.7)	0.329
Vit B6 (mg)	314.1 (105.7)	305.5 (94.4)	321.9 (116.7)	0.842
Biotin (ug)	117.7 (76.2)	135.7 (87.3)	101.2 (61.7)	0.162
Folic Acid (ug)	205.4 (81.4)	199.1 (86.1)	211.2 (78.4)	0.565
Vit B12 (ug)	636.2 (273.9)	657.5 (321.6)	616.6 (227.5)	0.842
Ascorbic Acid (mg)	451.6 (244.3)	431.2 (248.7)	470.2 (244.2)	0.647
Vit A (ug)	239.5 (93.4)	250.7 (86.9)	229.3 (99.8)	0.549
Vit D (ug)	64.9 (65.9)	69.5 (80.5)	60.6 (50.6)	0.897
Vit E (mg)	228.5 (143.7)	191.8 (100.7)	262.1 (169.4)	0.065
***Food Group: Weekly Consumption***				
Dairy Products (n°. consumed/week)	21.2 (13)	22.7 (14.9)	19.9 (11.1)	0.487
Eggs, Meats and Fish (n°. consumed/week)	23.3 (9.3)	21.8 (10.9)	24.6 (7.5)	0.188
Vegetables (n°. consumed/week)	25.3 (12.9)	23.7 (14.2)	26.8 (11.8)	0.188
Fruits (n°. consumed/week)	22.4 (17.7)	19 (18.4)	25.4 (16.8)	0.086
Legumes and Cereals (n°. consumed/week)	6.6 (4.9)	5.9 (3.9)	7.2 (5.7)	0.687
Sugars and ultra-processed products (n°. consumed/week)	53.9 (22.4)	45.5 (14.9)	61.6 (25.4)	0.03
***Weekly food record***				
R24-weekly (n°. of symbiotic foods consumed/week)	24.4 (7.8)	20.6 (7.8)	27.8 (6.2)	0.001
R24-monthly (n°. of symbiotic foods consumed/week)	97.7 (31.4)	82.6 (31.3)	111.4 (25)	0.001
R24-trimestral (n°. of symbiotic foods consumed/week)	293.1 (94.3)	247.9 (94.1)	334.3 (75)	0.001
***Anthropometric Profile***				
Weight (kg)	81.4 (17.6)	76.6 (18)	85.7 (16.3)	0.086
Waist circumference (cm)	101.9 (17)	97.6 (21)	105.7 (11.5)	0.312
BMI (kg/m^2^)	28.5 (5)	27.5 (5.2)	29.5 (4.8)	0.307
WHtR	0.6 (0.1)	0.6 (0.1)	0.6 (0.0)	0.518
Height (cm)	168.5 (9.2)	166.4 (10.7)	170.3 (7.4)	0.245
***Therapeutic Variables***				
N of associated antipsychotic	1.3 (0.5)	1.3 (0.5)	1.3 (0.4)	0.597
DDD antipsychotics (mg)	271.4 (242.5)	286.7 (222.3)	257.4 (242.5)	0.458

RDA: Recommended Dietary Allowance; Ca: calcium; Mg: magnesium: P: phosphorus; Na: sodium; K: potassium; Fe: iron; Cu: copper; Zn: zinc; Mn: manganese; I: iodine; Se: selenium; Vit.B6: vitamin B6; Vit.B12: vitamin B12; Vit.A: vitamin A; Vit.D: vitamin D; Vit.E: vitamin E; Food Group-Weekly Consumption: Calculation of average consumption of main foods (weekly) by food group; Weekly food record: weekly determination of foods with high symbiotic content in adherence to established dietary plan; R24-weekly: average (weekly) calculation of the main foods and dishes consumed according to the established dietary plan; R24-monthly: average (monthly) calculation of the main foods and dishes consumed according to the established dietary plan; R24-quarterly: average (quarterly) calculation of the main foods and dishes consumed according to the established dietary plan BMI: body mass index; WHtR: waist-to-height ratio; Antipsychotic DDD: defined daily dose antipsychotics.

**Table 3:** Modifications in allocation groups: control group and experimental group.

	Total (*n* = 44)			Control Group (*n* = 21)			Intervention Group (*n* = 23)			*p* *	*p* **
Variables	Basal	6 Months	*p*	Basal	6 Months	*p*	Basal	6 Months	*p*		
***Macronutrients*** (RDA)											
Energy (%)	177.4 (48.4)	128.2 (31.7)	<0.001	182 (47.3)	130.9 (37.8)	0.001	173.2 (50.1)	125.8 (25.4)	<0.001	0.329	0.647
Proteins (g)	432 (152.5)	328.4 (116.7)	<0.001	443.2 (159.6)	311.1 (134.1)	0.003	421.9 (148.6)	344.2 (98.6)	0.011	0.597	0.209
Lipids (g)	207.8 (63.1)	143.2 (39.2)	<0.001	212.4 (68.2)	149.6 (47)	0.002	203.5 (59.4)	137.3 (30.2)	<0.001	0.716	0.245
Saturated fatty acids (g)	392.3 (222.2)	251.6 (93.8)	<0.001	414.9 (260.6)	252.4 (99.4)	0.002	371.7 (183.9)	250.9 (90.7)	0.008	0.698	0.897
Monounsaturated fatty acids (g)	147.7 (60.9)	108.7 (40.2)	<0.001	140.9 (63.6)	103.1 (35.9)	0.006	154 (59)	113.9 (43.9)	0.018	0.613	0.418
Polyunsaturated fatty acids (g)	138.9 (103.7)	120.6 (115.8)	0.264	145.1 (131.3)	144.6 (160)	0.985	133.2 (72.8)	98.7 (43.3)	0.095	0.716	0.733
Cholesterol (mg)	247.2 (135.8)	173.1 (80.7)	0.001	224.6 (70.3)	160.1 (76.7)	0.002	267.9 (175)	185 (84.2)	0.027	0.787	0.285
Carbohydrates (g)	159.6 (53.4)	118.7 (35.2)	<0.001	161.8 (48.2)	120.3 (38.5)	0.003	157.5 (58.7)	117.3 (32.6)	0.004	0.518	0.897
Oligosaccharides (g)	303.4 (205.7)	204.8 (98.6)	0.005	342.7 (203.8)	214 (113.4)	0.015	267.5 (205.2)	196.5 (84.6)	0.134	0.088	0.805
Polysaccharides (g)	123.2 (74.6)	96.6 (40.7)	0.019	139.8 (87.2)	89.1 (44.4)	0.003	108 (58.8)	103.5 (36.6)	0.762	0.378	0.177
Fibre (g)	215.1 (124.8)	185.8 (111)	0.229	196 (130.5)	175.8 (116.8)	0.560	232.5 (119.5)	194.8 (107.3)	0.288	0.245	0.431
***Micronutrients*** (RDA)											
Ca (mg)	181 (68.8)	142 (56)	0.004	194.1 (64.7)	142.8 (64.6)	0.02	169.2 (71.2)	141.3 (48.2)	0.099	0.124	0.751
Mg (mg)	292.8 (95.4)	238.2 (102.7)	0.002	285.3 (88.8)	220.2 (116.8)	0.013	299.7 (102.6)	254.7 (87.3)	0.07	0.823	0.118
P (mg)	352.9 (107.7)	270.9 (77.8)	<0.001	372.7 (123.5)	265.6 (88.2)	0.003	334.8 (89.9)	275.7 (68.6)	0.009	0.209	0.318
Na (mg)	310.8 (93.6)	201 (60.7)	<0.001	320.9 (112.4)	200.5 (67.6)	0.001	301.5 (73.7)	201.5 (55.2)	<0.001	0.630	0.860
K (mg)	236 (83.8)	195.5 (84.4)	0.007	232.4 (90.2)	178.4 (87.7)	0.03	239.2 (79.5)	211 (80.1)	0.120	0.664	0.136
Fe (mg)	216 (70.5)	169.5 (57.6)	<0.001	211.1 (64.1)	166.9 (66.3)	0.008	220.5 (77.1)	171.8 (49.8)	0.003	0.953	0.953
Cu (mg)	173.7 (91.2)	154.8 (73.8)	0.170	170 (118.9)	133.9 (74.5)	0.132	177 (58.1)	173.8 (69.3)	0.829	0.184	0.08
Zn (mg)	260.4 (101.3)	202.3 (76.4)	0.003	251.2 (83.4)	206.3 (96.5)	0.118	268.7 (116.6)	198.6 (53.9)	0.013	0.916	0.565
Mn (mg)	714.7 (595.6)	632.8 (437.2)	0.413	575.9 (360.2)	495.5 (376.9)	0.290	841.3 (735)	758.3 (458.3)	0.648	0.264	0.028
I (ug)	252.7 (102.1)	194.9 (91.9)	0.009	278.4 (120.9)	204.2 (114.7)	0.061	229.2 (76.7)	186.4 (66.3)	0.063	0.177	0.953
Se (mg)	501.4 (229)	392.4 (147.6)	0.004	471 (203)	360.4 (136.2)	0.034	529.2 (251.7)	421.6 (154.4)	0.055	0.518	0.162
Thiamine (mg)	261.4 (81.4)	209.6 (65.2)	<0.001	261 (74.1)	203.5 (76.8)	0.006	261.7 (89.2)	215.2 (53.7)	0.024	0.769	0.503
Riboflavin (mg)	289.2 (92.7)	237.2 (72)	0.005	299.5 (92.1)	230.3 (79.9)	0.019	279.8 (94.4)	243.5 (65)	0.135	0.226	0.534
Niacin (mg)	388.5 (116.3)	318 (98.4)	0.001	392.7 (113)	295.9 (114.4)	0.002	384.7 (121.6)	338.1 (78.3)	0.104	0.503	0.107
Pantothenic acid (mg)	97.3 (44.6)	82.3 (32.2)	0.051	103.6 (46.9)	73 (29.4)	0.019	91.6 (42.7)	90.8 (32.8)	0.929	0.329	0.08
Vit B6 (mg)	314.1 (102.7)	262.9 (97.8)	0.007	305.5 (94.4)	244.2 (104.5)	0.025	321.9 (116.7)	279.9 (90.2)	0.128	0.842	0.155
Biotin (ug)	117.7 (76.2)	104.6 (73.6)	0.363	135.7 (87.3)	93.5 (62.3)	0.07	101.2 (61.7)	114.7 (82.7)	0.429	0.162	0.296
Folic Acid (ug)	205.4 (81.4)	174.8 (69.9)	0.02	199.1 (86.1)	158.3 (64.7)	0.057	211.2 (78.4)	189.9 (72.4)	0.195	0.565	0.142
Vit. B12 (ug)	636.2 (273.9)	475.6 (198.3)	0.002	657.5 (321.6)	449.5 (183.2)	0.021	616.6 (227.5)	499.4 (212.3)	0.04	0.842	0.549
Ascorbic Acid (mg)	451.6 (244.3)	429 (206.7)	0.529	431.2 (248.7)	383.2 (226.1)	0.292	470.2 (244.2)	470.8 (287.3)	0.992	0.647	0.254
Vit. A (ug)	239.5 (93.4)	219 (80.1)	0.212	250.7 (86.9)	208.6 (75.7)	0.079	229.3 (99.8)	228.6 (84.4)	0.974	0.549	0.366
Vit. D (ug)	64.9 (65.9)	52.4 (56.1)	0.13	69.5 (80.5)	56.4 (78.4)	0.201	60.6 (50.6)	48.8 (23.1)	0.361	0.897	0.445
Vit. E (mg)	228.5 (143.7)	156.4 (52.8)	0.002	191.8 (100.7)	146.9 (64.3)	0.059	262.1 (169.4)	165.1 (39.1)	0.014	0.065	0.062
***Food Group: Weekly Consumption***											
Dairy Products (n°. consumed/week)	21.3 (13)	22.4 (11.9)	0.625	22.7 (14.9)	24.4 (13.8)	0.670	19.9 (11.1)	20.5 (9.8)	0.822	0.487	0.316
Eggs, Meats and Fish (n°. consumed/week)	23.3 (9.3)	20 (7.6)	0.097	21.8 (10.9)	22.1 (8.7)	0.927	24.6 (7.5)	18.1 (5.9)	0.009	0.188	0.09
Vegetables (n°. consumed/week)	25.3 (12.9)	23.8 (11.1)	0.517	23.7 (14.2)	23 (12.9)	0.848	26.8 (11.8)	24.6 (9.4)	0.407	0.188	0.284
Fruits (n°. consumed/week)	22.4 (17.7)	18.2 (14.4)	0.074	19 (18.4)	15.3 (14)	0.276	25.4 (16.8)	20.8 (14.6)	0.165	0.086	0.148
Legumes and Cereals (n°. consumed/week)	6.6 (4.9)	7.2 (4.2)	0.380	5.9 (3.9)	6.4 (2.5)	0.562	7.2 (5.7)	7.9 (5.2)	0.522	0.687	0.661
Sugars and ultra-processed products (n°. consumed/week)	53.9 (22.4)	57.4 (28.4)	0.505	45.5 (14.9)	67.2 (34.1)	0.006	61.6 (25.4)	48.4 (18.5)	0.03	0.03	0.037
***Weekly food record***											
R24-weekly (n°. of symbiotic foods consumed/week)	24.4 (7.8)	-	-	20.6 (7.8)	-	-	27.8 (6.2)	-	-	0.001	-
R24-monthly (n°. of symbiotic foods consumed/week)	97.7 (31.4)	-	-	82.6 (31.3)	-	-	111.4 (25)	-	-	0.001	-
R24- quarterly (n°. of symbiotic foods consumed/week)	293.1 (94.3)	-	-	247.9 (94.1)	-	-	334.3 (75)	-	-	0.001	-
***Anthropometric Profile***											
Weight (kg)	81.4 (17.6)	78.7 (16.2)	<0.001	76.6 (18)	75.8 (17.7)	0.382	85.7 (16.3)	81.3 (14.6)	<0.001	0.086	0.275
Waist circumference (cm)	101.9 (17)	101.6 (12.5)	0.898	97.6 (21)	101.2 (13.5)	0.322	105.7 (11.5)	102.1 (11.7)	<0.001	0.397	0.981
BMI (kg/m^2^)	28.5 (5)	27.6 (4.7)	<0.001	27.5 (5.2)	27.2 (5.3)	0.323	29.5 (4.8)	27.9 (4.3)	<0.001	0.307	0.869
WHtR	0.6 (0.09)	0.6 (0.07)	0.932	0.6 (0.12)	0.6 (0.08)	0.345	0.6 (0.06)	0.6 (0.06)	<0.001	0.597	0.378
***Therapeutic Variables***											
N° of associated antipsychotic	1.3 (0.5)	1.2 (0.4)	0.262	1.38 (0.49)	1.28 (0.46)	0.329	1.3 (0.47)	1.26 (0.44)	0.575	0.597	0.855
DDD antipsychotics (mg)	271.4 (242.5)	241.2 (226.7)	0.108	286.7 (222.3)	260.5 (221.5)	0.230	257.4 (263.7)	247.4 (225.9)	0.301	0.458	0.789

*p*: intragroup statistical significance; *p* *: baseline intergroup statistical significance; *p* **: 6 months intergroup statistical significance; RDA: Recommended Dietary Allowance Ca: calcium; Mg: magnesium: P: phosphorus; Na: sodium; K: potassium; Fe: iron; Cu: copper; Zn: zinc; Mn: manganese; I: iodine; Se: selenium; Vit.B6: vitamin B6; Vit.B12: vitamin B12; Vit.A: vitamin A; Vit.D: vitamin D; Vit.E: vitamin E; Food Group-Weekly Consumption: Calculation of average consumption of main foods (weekly) by food group; Weekly food record: weekly determination of foods with high symbiotic content in adherence to established dietary plan; R24-weekly: average (weekly) calculation of the main foods and dishes consumed according to the established dietary plan; R24-monthly: average (monthly) calculation of the main foods and dishes consumed according to the established dietary plan R24-quarterly: average (quarterly) calculation of the main foods and dishes consumed according to the established dietary plan BMI: body mass index; WHtR: waist-to-height ratio; Antipsychotic DDD: defined daily dose antipsychotics.

## Data Availability

The collected data that support the findings of this study are available on reasonable request from the corresponding author.

## Trial Registration

The referral Research Ethics Committee approved the study protocol in November 2019 (reg. 468) and retrospectively registered at clinicaltrials.gov (NCT0436366401. First Submitted: 28 April 2020).
